# Ethanol-Modified, Supercritical CO_2_ Extraction of Persimmon (*Diospyros kaki* L.) Side Streams for Innovative Bioactive and Antimicrobial Formulations: A Pilot Level Proof-of-Concept and Green Chemistry Evaluation

**DOI:** 10.1021/acsomega.6c05435

**Published:** 2026-09-16

**Authors:** Albert Sebastià, Manuel Salgado-Ramos, Noelia Pallarés, Samantha López-Contreras, Sandra Garrigues, Juan A. Tamayo-Ramos, Raquel Lucas-González, Juana Fernández-López, Francisco J. Barba

**Affiliations:** † Research Group in Innovative Technologies for Sustainable Food (ALISOST), Department of Preventive Medicine and Public Health, Food Science, Toxicology and Forensic Medicine, Faculty of Pharmacy and Food Sciences, 16781 Universitat de València, Avenida Vicent Andrés Estellés, 22, 46100 Burjassot, Valencia, Spain; ‡ Food Biotechnology Department, 83071 Instituto de Agroquímica y Tecnología de Alimentos (IATA), Consejo Superior de Investigaciones Científicas (CSIC), 46980 Paterna, Valencia, Spain; § Research Group in Innovations in Food Products (IPOA), Institute for Agri-Food and Agri-Environmental Research and Innovation, Miguel Hernández University (CIAGRO-UMH), Carretera Beniel Km 3.2, 03312 Orihuela, Alicante, Spain; △ CIBER de Enfermedades Infecciosas (CIBERINFEC), Instituto de Salud Carlos III, C/Monforte de Lemos 3–5. Pabellón 11. Planta 0, 28029 Madrid, Spain

## Abstract

This study addresses the sustainable valorization of persimmon (*Diospyros kaki* L.) side streams via process intensification through supercritical CO_2_ technology (Sc-CO_2_) (35 MPa, 50 °C, 120 min). Namely, the ethanol proportion in the supercritical stream and different levels of operation (LoOP) were tested. In particular, the process at a pilot LoOP (2%-ethanol-doped, 2 kg of sample) enabled the production of a lipophilic extract with enhanced nutritional and biological activity, making it a promising candidate for further research in nutraceutical formulations. More deeply, the extract demonstrated a noted antimicrobial activity against bacteria (*Escherichia coli*, *Bacillus subtilis*) and filamentous fungi (*Penicillium expansum*), with minimum inhibitory concentrations (MIC) ranging from 0.8-7-8 μL/mL that were similar as the benchmark (oregano extract). In addition, the crude displayed a noted Fe content (85.8 mg/100 g extract), with a relevant presence of Mg as well (up to 52% RDI in 100 g extract). The environmental sustainability of the process (pilot level, 2%-ethanol-doped) was assessed through green chemistry metrics. Namely, the protocol displayed 45.9 score in the eco-scale, and a ten-fold reduction for PMI or CEF compared to bench scale experiments (2%-ethanol). Overall, this work highlights the efficiency of an advanced methodology to produce a bioactive extract from persimmon culls that represents a promising candidate for the development of nutraceuticals.

## Graphical abstract

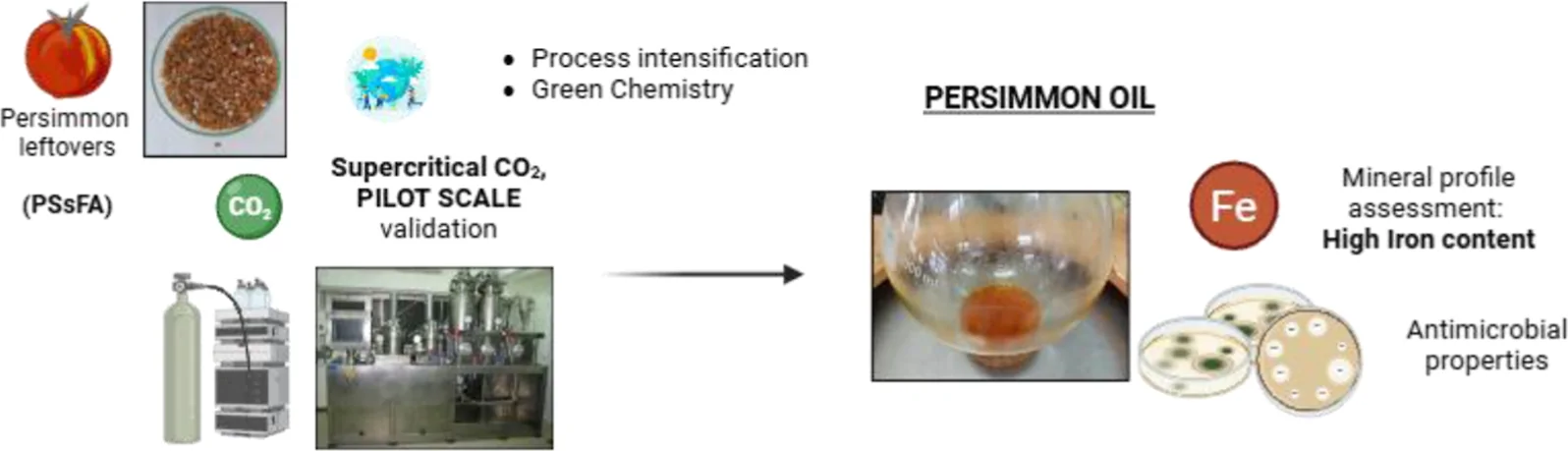

## 1 Introduction

Persimmon (*Diospyros kaki* L.) is a widely consumed fruit with increasing industrial relevance due to its sensory attributes and high content of bioactive compounds. Beyond fresh consumption, persimmons are processed into juices, dried products, and fermented beverages.^1,2^ This entails the generation of significant amounts of side streams, particularly peel and fruit apex residues.^3,4^ These fractions contain a notable content of dietary fiber, minerals, and lipophilic compounds, mainly carotenoids and phenolics, which are associated with antioxidant and functional properties.^5,6^ Furthermore, persimmon pulp contains approximately 80% water, low fat content, and sugars, mainly glucose and fructose, while the peel is significantly rich in fiber, minerals, and bioactive compounds.^5,7^ This uneven distribution of valuable compounds makes persimmon side-streams especially attractive for valorization.

However, strict commercial standards related to size, shape, and ripeness result in substantial post-harvest losses, with more than 25% of production being discarded or underutilized.^8,9^ This loss represents both an environmental concern and an opportunity for the development of value-added products.

Among the bioactive compounds present in persimmon residues, carotenoids and polyphenols have attracted particular attention because of their antioxidant capacity and technological functionality.^10^ In addition to their role as radical scavengers, these compounds can exhibit antimicrobial activity against bacteria, yeasts, and filamentous fungi, making them promising natural alternatives to synthetic preservatives. The antimicrobial mechanisms involve disruption of cell membranes, enzyme inhibition, and metal ion chelation.^11^

Despite this potential, the main challenge resides in developing novel, sustainable protocols to recover bioactive substances. The high sugar content and structural complexity of the matrix may hinder mass transfer and extraction efficiency, while carotenoids are highly sensitive to oxidation and thermal degradation.^12^ These factors highlight the need for extraction techniques capable of preserving compound integrity while ensuring efficient recovery in a sustainable way.

As a substitute for traditional extraction methods, process intensification through non-conventional technologies has emerged over the last decades as a novel, green strategy.^13^ In particular, supercritical carbon dioxide (Sc-CO_2_) represents a suitable solution for recovering lipophilic bioactive compounds, allowing to avoid the use of organic solvents such as hexane or chloroform. More specifically, Sc-CO_2_ combines gaslike diffusivity and liquidlike solvating power, allowing efficient penetration into solid matrices and selective extraction of targeted compounds. The efficiency of Sc-CO_2_ is strongly influenced by operating parameters such as pressure, temperature, extraction time, and co-solvent addition, which affect solvent density, solubility, and mass transfer phenomena.^14,15^

Although Sc-CO_2_ has been widely studied at the laboratory scale, its implementation at larger levels of operation still poses a real challenge. Particularly for persimmons, a highly relevant crop in Valencian agriculture, limited literature has reported the use of Sc-CO_2_ at a high level, to the best of our knowledge. The transition from lab- to pilot-scale introduces additional challenges related to mass transfer limitations, solvent distribution, process efficiency, and environmental impact.^16^ In addition, sustainability evaluations, for instance, through green chemistry indicators (environmental factor, process mass intensity, or eco-scale), become essential to assess the real scope and the industrial feasibility of the process.^17^

To provide novel solutions that address the above-mentioned issues, this study reports the development of a novel bioactive extract from persimmon culls that paves the way for further investigations in the search for novel nutraceutical products. Supercritical CO_2_ is presented as a process intensification strategy to be proven at the pilot level, filling the gap between lab- and big-scale processes. The adjustment of doping with GRAS solvents (ethanol, EtOH) in the supercritical stream as well as the assessment of various levels of operations (LoOP) prior to the pilot level confirmed the suitability of the methodology. In this way, novel advances in the state-of-the-art were provided regarding novel food and antimicrobial formulations, Sc-CO_2_ processing of persimmons, and large-scale operations. The environmental footprint of the methodology was evaluated with different green chemistry metrics, presenting a comprehensive outlook on sustainability and its further implementation in biorefineries.

## 2 Materials and Methods

### 2.1 Samples and Reagents

Persimmon samples were provided by the technological center AINIA (Paterna, Spain). In this study, persimmon peels (PPs) combined with fruit apex (FA) residues (PPsFA) were considered for this study. Feedstock preparation took place in AINIA (Paterna, Spain) and included the peeling of discarded fresh fruit, drying at low temperature (45 °C) until constant mass, and a final blade-milling step (particle size distribution ø> 1.6 mm).

For the reactants, absolute ethanol (EtOH, CAS N°: 64-17-5) and sodium carbonate (Na_2_CO_3_, >99%, CAS N°: 497-19-8) were provided by VWR Chemicals (Rosny-sous-Bois, France); CO_2_ (99%, CAS N° 124-38-9) was supplied by Carburos Metálicos (Massalfassar, Valencia, Spain) in 50 L cartridges; Folin–Ciocalteu reagent, gallic acid (97.5–100%, CAS N°: 149-91-7), ABTS (2,2′-azino-bis-3-ethylbenzothiazoline-6-sulfonic acid, 98%, CAS N°: 30931-67-0), Trolox (6-hydroxy-2,5,7,8-tetramethylcroman-2-carboxylic acid, 97%, CAS N°: 53188-07-1), CDCl_3_ (99.8%, with 0.04% TMS, CAS N°: 865-49-6), and 2-methyltetrahydrofuran (>99.5%, 150–400 ppm BHT, CAS N°: 96-47-9) were supplied by Sigma-Aldrich (Steinheim, Baden-Württemberg, Germany). The concentrated standards of macro/trace element titrisol (Mg, Ca, P, Fe, Zn, Se) and nitric acid (HNO_3_) were purchased from Merck (Darmstadt, Germany).

### 2.22.2 Supercritical Fluid Extraction (SFE)

#### 2.2.1 Lab-Scale Experiments

For lab scale, SFE was performed in a JASCO supercritical equipment (Jasco Analítica Spain, S.L.), located at the ALISOST lab, at the Faculty of Pharmacy and Food Sciences at the University of Valencia (Valencia, Spain). This instrument allowed us to operate by continuous flow conditions. Technical specifications of each hold are:

- Isocratic CO_2_ pump (PU-4387), with a flow ranged from 5 to 40 mL/min, adjustable in 0.01 mL/min increasements, 50 MPa top pressure, and an extraction system for pulses.
- Refrigerating recirculating cooler (JULABO FL 1201) for pump cooling, with a temperature range of −20–40 °C, flow range of up to 23 L/min, and a cooling refrigeration capacity of 1.2 kW at 20 °C.
- Isocratic organic modifier pump (PU-4086, HPLC), with a flow ranging from 0.001–10 mL/min, adjustable in 0.01 mL/min increments, and a 70 MPa top pressure.
- Thermostatic oven for glass reaction (CO-4065), with a temperature range from 4 to 90 °C.
- Thermostatic system by Peltier and temperature transference by air flow.
- Pressure regulator (BP-4340).
- Stainless steel, extraction glasses with different capacities: 5 mL (803560–5 ML) and 50 mL (803560–50 ML).

Two methodologies were applied at this level of operation. Firstly, as a proof-of-concept in the search for optimal conditions, a 5 mL extraction stainless-steel reactor was selected. A total of 3.5 g of crushed persimmon peels + fruit apex (PPsFA) were loaded. Due to the high sugar content in this matrix, a minor proportion of an inert material (sepiolite, 17%) was combined with the pertinent charge of PS to facilitate crushing and milling, as well as the subsequent processing. The setting of the key experimental variables (pressure, temperature, and extraction time) was conducted by means of a Box-Behnken design through the ChromNav Control Center software (Table S1). Absolute flow was selected at 16 mL/min, based on previous studies that reported a supercritical CO_2_ extraction.^18^ A minimum proportion of a doping cosolvent (EtOH, 10%) was initially introduced according to our previous study.^18^ Optimal conditions provided by the software based on the oil yield (Table S1) were found as 35 MPa pressure, 50 °C temperature, and 120 min time. Three replicates were carried out experimentally to confirm this optimization. After that, a minor reduction of EtOH proportion (up to 4%) was applied. Experiments were performed by triplicate as well.

Optimal conditions at small scale were then reproduced to bench scale (35 g). At this point, minor modifications were also applied in terms of doping proportion (2% EtOH), while absolute flow was increased up to 40 mL/min. Both were made to try to adjust the experimental conditions for a pilot scale extraction as much as possible. The main consideration was to reduce the EtOH flow to attenuate environmental footprint due to the use of organic solvents. Experiments were performed by triplicate and expressed as mean ± SD.

For both levels, two extraction products were recovered. The remaining solid fraction, defatted after the supercritical CO_2_ processing, was stored in plastic jars and protected from light. It was kept at room temperature and then weighed after EtOH evaporation to calculate extraction yield (eq 1). The liquid extract (an EtOH-rich crude) was continuously collecting and storing in amber glass (Pyrex) collector bottles during the supercritical extraction. The EtOH was evaporated using a rotary steam (Büchi rotavapor R-200) and the resulting extract stored in an amber-colored glass vial. It was maintained at 4 °C for further assays and served to calculate oil and defatting yield (eq 1).

#### 2.2.2 Pilot-Scale Extraction

Aiming at implementing the process in a biorefinery context, a pilot scale extraction was conducted as a proof-of-concept to test the real scope of the previous methodology at lab-scale. Previously optimized conditions at bench scale in respecting of EtOH doping (2% of the Sc-CO_2_ stream) were considered. In parallel, two proofs-of-concept were executed by modifying EtOH stream (0%, 4%) to evaluate process efficiency. Experiments for 2–4% were conducted following the optimized parameters at lab scale: P: 35 MPa, T: 50 °C. Contrarily, in absence of co-solvent, 40 MPa pressure were applied to facilitate the release of the extract.

2 kg of PPsFA (in combination with 17% sepiolite) was processed in 20 L reactors for 0–2% experiments; for the 4%-EtOH-doped system, only 1 kg of PSsFA was processed to keep an acceptable solid:liquid ratio. Processing time was adjusted based on a kinetic curve that displays maximum efficiency in terms of defatting (5 h). Absolute flow or other technical setups required at this level of operation were considered. The extraction was performed once (0, 2, and 4%) because of availability and cost issues in the pilot plant.

### 2.3 Extraction Performance

Process efficiency was measured by means of extraction, oil, and defatting yield according to eqs 1–3. Particularly for defatting, the amount of lipophilic fraction present in the PPsFA was considered, attending to the initial feedstock charged in the reactor (eq 3). Lipophiles percentages were determined by means of a Soxhlet extraction with refluxed 2-methyltetrahydrofuran (2-MeTHF) for 6 h.

1 $$Extraction\;yield\left(\%\right)=\frac{mo\left(PPsFA\right)-mass\;solid\;after\;extraction\left(g\right)}{mo\left(PPsFA\right)\left(g\right)}\times 100$$

2 $$Oil\;yield\left(\%\right)=\frac{mass\;of\;oil\left(g\right)}{mo\left(PPsFA\right)\left(g\right)}\times 100$$

3 $$Defatting\left(\%\right)=\frac{mass\;of\;oil\left(g\right)}{lipophiles\;content\;present\;in\;mo\left(PPsFA\right)\left(g\right)}\times 100$$

### 2.4 Chemical Characterization of the Recovered Oil: Lab-, Bench-, and Pilot Scale

The quantitative determination of the pigments and bioactive compounds present in the extract after SFE treatments (lab-, bench-, and pilot scale) was carried out using UV/VIS spectrophotometry through absorbance measurements. It was determined in a FLUOstar OMEGA plate reader (BMG Labtech GmbH, Ortenberg, Germany) in 96-well plates. Table 1 summarizes the samples evaluated.

**Table 1:** Extracts Chemically Evaluated in Preliminary Assays

supercritical CO_2_ device	ethanol doping
lab scale (optimal conditions) (3.5 g PPsFA)	10% and 4%
bench scale (35 g)	2%
pilot scale (2 kg)	0%, 2%, and 4%

#### 2.4.1 Total Carotenoids (TC), Total Phenolic Content (TPC), and Total Antioxidant Capacity (TAC)

TC were determined in a 96-well plate through absorbance measurement at 648, 664, and 470 nm following the previously reported literature,^19^ with some modifications. Dried extracts were diluted in the appropriate amount of EtOH, and the extract charged in at least 3 wells to assure reproducibility. TC were expressed as the sum of carotenoids (TC._x+c_) and were calculated through eqs 4–6 considering other pigments (chlorophyll A (Ca) and B (Cb)):

4 $$C_{a}\left(\mu g/ml\right)=13,36{\;A}_{664,1}-5,19{\;A}_{648,6}$$

5 $$C_{b}\left(\mu g/ml\right)=27,43{\;A}_{648,6}-8,12{\;A}_{664,1}$$

6 $$TC_{x+c}\left(\mu g/ml\right)=\left(1000{\;A}_{470}-2,13\;C_{a}-97,64{\;C}_{b}\right)/209$$

TPC was measured through the Folin–Ciocalteu test at 765 nm and expressed as mg gallic acid equivalents (GAE)/g extract. TEAC (Trolox Equivalent Antioxidant Capacity) assay was carried out by means of absorbance measurement at 734 nm due to the presence of the ABTS + radical. Trolox was used as standard, so that results were referred as μmol Trolox Equivalent (TE)/g extract. Both protocols were developed following the methodology previously described,^20^ with some modifications.^21^

For all these methods, experiments were performed by triplicate and the results expressed as mean ± standard deviation (SD).

#### 2.4.2 Inductively Coupled Plasma Mass Spectrometry (ICP–MS) Mineral Determination (Pilot Scale)

For the determination of minerals, a suspension of the persimmon crude in EtOH was prepared at a concentration of 1 mg/mL. The sample underwent a digestion process in a microwave oven, following the method described by Bernabeu et al.,^22^ with some modifications. For this purpose, HNO_3_ (14 M, 4 mL) and H_2_O_2_ (30% v/v, 1 mL) were added to the samples. Digestion was carried out at 800 W and 180 °C for 15 min. The samples were then taken and allowed to cool to room temperature. After extracting the nitrogen, the samples were filtered, and the volume was adjusted with distilled water. Next, the determination of minerals was carried out by ICP–MS in an Agilent equipment, model 7900. The conditions applied were as follows: carrier gas flow at 1.07 L/min, helium (He) as reactive gas, high-frequency emission power at 1550 W, argon gas flow (Ar) at 15.0 L/min, nebulizer pump speed at 0.10 rps, and radio frequency matching at 1.80 V. The determination was carried out in triplicate, and results expressed as mean ± SD.

#### 2.4.3 Nuclear Magnetic Resonance (NMR) (Conventional Soxhlet Extraction)

^1^H NMR analysis was conducted on a Bruker AV400 spectrometer located at SCSIE (University of Valencia) which operates at a frequency of 400 MHz for ^1^H nucleus. ^1^H NMR spectrum was executed with a broadband inverse (BBI) 5 mm multinuclear probe (^1^H/BB), equipped with a *z*-axis field gradient and automatic frequency tuning, proton channel, and a broadband multinuclear channel. For sample preparation, 30 mg of extract were suspended in 600 μL of d-chloroform (CDCl_3_) that acted as solvent. The crude was introduced in a 5 mm NMR tube. No prior filtration was required due to an effective solubilization of the crude. Experimental parameters were set at 16 scans, and 5 s D1, which corresponded to an acquisition time of 1 min.

#### 2.4.4 Antimicrobial Activity (Pilot Scale)

The antimicrobial activity of the persimmon lipophilic extract was tested against the bacteria *Bacillus subtilis* CECT 498 and *Escherichia coli* JM109; the yeasts *Saccharomyces cerevisiae* BY4741 and *Zygosaccharomyces bailii* J81; and the filamentous fungi *Penicillium expansum* A72 and *Aspergillus flavus* RB9. Bacteria, yeasts, and filamentous fungi were routinely cultured on Luria–Bertani (LB) agar, Yeast Extract Peptone Dextrose (YPD) agar, and Potato Dextrose Agar (PDA) plates, respectively.

Growth inhibition assays were performed in 96-well flat-bottom microtiter plates (Nunc Delta Surface, Thermo Scientific) following the broth microdilution method according to (EUCAST) & (ESCMID), 2003,^23^ with minor modifications. Both persimmon and oregano oils, the last used as positive control of antimicrobial activity,^24^ were diluted in two-fold serial dilutions using 10% (v/v) of dimethyl sulfoxide (DMSO) as a solvent (2× concentrated). The oil extract concentration ranges were: 0.006–12.5 μL/mL for bacteria, 0.003–1.95 μL/mL for yeasts and, 0.031–31.25 μL/mL for filamentous fungi. The bacterial and yeast inoculums (2× concentrated) were 1 × 10^6^ CFU/mL, obtained from a pre-culture in the nutrient-rich LB and YPD media, respectively. In case of filamentous fungi, the inoculum was 5 × 10^4^ spores/mL, which was adjusted using a Neubauer hemocytometer. Bacterial, yeast, and fungal inoculums were prepared 2× concentrated in diluted media (20% LB, 40% YPD, 10% (w/v) Potato Dextrose Broth, respectively). Additionally, yeast and fungal inoculums contained 0.02% (w/v) chloramphenicol to avoid bacterial contamination.

Each well contained a total volume of 100 μL in which 50 μL of oil dilutions were mixed with 50 μL of each microorganism inoculum preparation. Samples were prepared in technical triplicates. Plates were statically incubated at 37 °C for bacteria, 30 °C for yeasts, and 25 °C for filamentous fungi. Optical density (OD) was measured at 600 nm using a SPECTROstar Nano spectrophotometer (BMG Labtech, Ortenberg, Germany) every 24 h. Dose–response curves were generated from measurements after 48 h in case of bacteria and yeasts, and 72 h in case of filamentous fungi. The experiments were repeated at least twice, employing triplicate biological replicates each time, and dose–response curves were displayed showing the mean ± standard deviation (SD) of the percentage of growth. The Minimum Inhibitory Concentration (MIC) is defined as the extract concentration that completely inhibited growth in all the experiments conducted. The Minimum Lethal Concentration (MLC) is defined as the lowest extract concentration needed to kill >90% of the microorganisms. The determination of MLC was only performed when a MIC was observed in case of the persimmon crude; in such cases, 50 μL from the corresponding wells were sub-cultured on rich-medium agar plates without the antimicrobial agent to determine the number of surviving cells (CFU/mL).

### 2.5 Sustainability Assessment: Green Chemistry Performance Metrics (GCPMs)

The environmental footprint of processes at bench and pilot scale was determined by means of green chemistry performance metrics (GCPMs). Indicators such as the complete environmental factor (EF) assessed the amount of waste generated throughout the process. Cumulative penalty points (CPPs) consider experimental setup, process efficiency, or safety and situate the protocol in an eco-scale (ES) from 0–100, with 100 being the greenest mark. The process was also evaluated in terms of the process mass intensity (PMI), process mass productivity (PMP), or reaction mass efficiency (RME). The methodology to define these metrics is based on previous studies^25^ and adapted to this work. A detailed protocol about the calculation of these GCPMs is included in the Supporting Information (Section S3).

### 2.6 Statistical Analyses

TPC, TEAC, and TC assays were conducted in triplicate, and results are expressed as mean ± standard deviation. Data processing and basic calculations were performed using Microsoft Excel (Microsoft Corp., Redmond, WA, USA).

For the optimization of supercritical fluid extraction (Sc-CO_2_) conditions, a Box–Behnken experimental design (BBD) was applied to evaluate the effects of three independent variables: pressure (MPa), temperature (°C), and extraction time (min). A second-order polynomial model was fitted to the experimental data

Y=β0+∑βiXi+∑βiiXi2+∑βijXiXj

where *Y* represents the response variable (oil yield and/or defatting yield), β_*0*_ is the intercept, β_*i*_, β*_i__i_*, and β*_i__j_* are the regression coefficients, and *X_i_* and *X_j_* are the independent variables.

The adequacy of the model was evaluated by analysis of variance (ANOVA), including determination of the coefficient of determination (R^2^), adjusted R^2^, and lack-of-fit tests. Statistical significance was established at p < 0.05. Response surface plots were generated to visualize the interaction effects between variables and determine optimal extraction conditions.

## 3 Results and Discussion

### 3.1 Feedstock Characterization: Determination of Lipophilic Content and Structural Determination

PSsFA was chemically characterized to quantify the lipophilic fraction, which is suitable for extraction in a supercritical CO_2_ environment. A solid–liquid extraction with a Soxhlet system was carried out by using 2-methyltetrahidrofuran as a substitute for traditional hexane.^26^ Total lipophiles were quantified by gravimetry, exhibiting 12.4% in PSsFA. To confirm the presence of lipophiles, the dried crude was chemically characterized by NMR (Figure 1).

**Figure 1:**
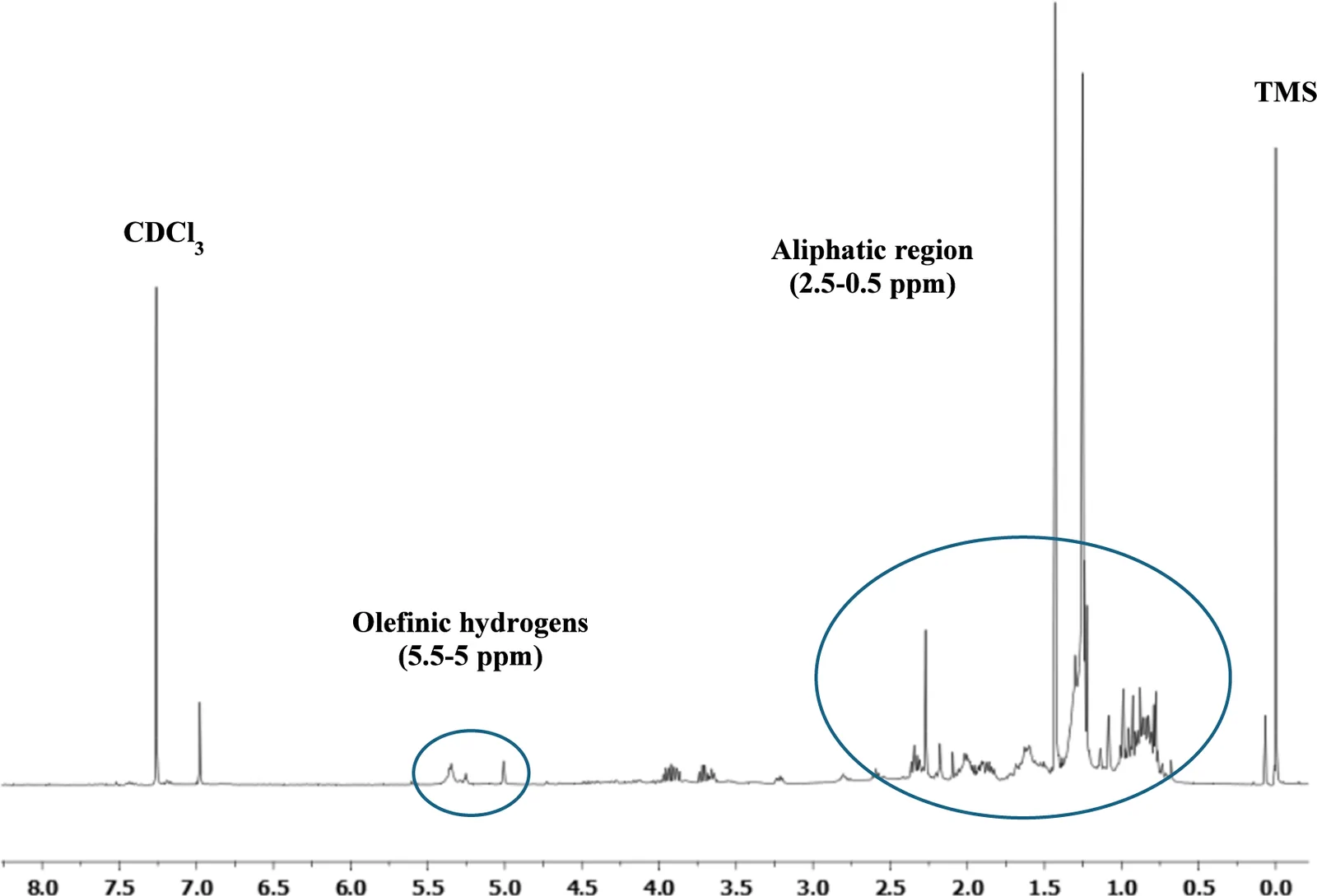
^1^H NMR spectrum (CDCl_3_, 400 MHz) of lipophilic fraction of persimmon peels combined with fruit apex residues (PSsAF) recovered with refluxed Me-THF (6 h).

As could be expected, a Me-THF extraction mainly leads to the recovery of low polar components. Particularly in persimmons, carotenoids are of relevance, with special emphasis on β-carotene.^4^ Thus, the ^1^H NMR spectrum displayed relevant signals at a region between 2.5–0.5 ppm, which can be associated with the aliphatic hydrogen of β-carotene. Namely, it should be noted the CH_3_- groups appearing at 1.5–1.2 ppm, as they are present in the molecule. Furthermore, the region between 5.5–5 ppm shows the presence of olefinic hydrogens in the structure, which are also present in β-carotene.

### 3.2 Process Optimization and Bioactivity Assessment: Lab-Scale Conditions

Supercritical fluid extraction of PSsFA was first applied at low scale (3.5 g, 5 mL reactor) to determine process efficiency based on extraction performance (oil and defatting yield). A Box-Behnken model served to determine preliminary experimental conditions for temperature, pressure, and extraction time (Table S1). Total flow was initially set at 16 mL/min, with a 10% cosolvent stream based on our previous studies.^18^

As can be observed in Table S1, process efficiency is notably conditioned by the three experimental parameters assessed, with a strong influence of extraction time (Figure S1). According to these outcomes, the oil yield increases from 3% (average of 30 min experiments) to 8–9% after 90 min, i.e., at 120 min. That implies a notable defatting at longer times, reaching values ranging from 73–80%. This increase in extraction yield efficiency can be attributed to increased contact of the supercritical solvent with the sample as the extraction time is extended.^27^

Furthermore, the optimal value for pressure was achieved at 35 MPa, close to the maximum value tested (40 MPa). Normally, high pressures entail an increase in the Sc-CO_2_ density that results in an enhanced solubility of the substances, which improves the extraction efficiency. Considering Table S1, defatting and oil yield reached prominent values (60–80%) when the extraction is conducted at 30–40 MPa, especially at longer time (120 min). Only when pressure is set at 20 MPa, process efficiency increased (77.7% defatting, Table S1, entry 4), although 120 min were required to achieve this value. These outcomes confirm the influence and trend of the model for PSsFA with respect to time and pressure (Figure S1).

At this stage of the study, it should be noted that the central point of the design (i.e., 50 °C, 75 min, 30 MPa), which served to validate the model, showed reproducibility in terms of oil and defatting yields (Table S1, **entries 3, 9** and **13**). However, these conditions did not afford the maximum efficiency and confirmed the strong influence of extraction time.

With respect to temperature, a balance between process efficiency and the breakdown of thermosensitive compounds should be considered. At this point and based on the data reported by the model (Figure S1), optimal values for temperature were achieved at 50 °C. As observed, extraction conducted at 40 °C did not lead to a high defatting (Table S1, **entries 1,8,12** and **15**), except when the experiment was carried out at 120 min and 30 MPa (**entry 15**), where process efficiency achieved a noticeable value (73.7% defatting). This confirmed the greater influence of pressure and time above temperature. Overall, the model shows that a correct management of pressure, temperature, and time variables is crucial to optimize extraction yields.

Optimal conditions were estimated once performance values for each experiment were provided (ChromNav Control Center software). For this study, maximal efficiency was predicted at 35.2 MPa pressure, 50.1 °C temperature, and 120 min extraction time, with expected oil and defatting yields of 8.9% and 72.1%, respectively. To validate these predictions, supercritical extraction was repeated under the same conditions, yielding an extraction efficiency of 23.9 ± 7.9%, an oil yield of 10.3 ± 0.7%, and a defatting yield of 82.9 ± 5.6%. Notably, the experimental results slightly exceeded the theoretical values, particularly for defatting. These outcomes confirm the validity of the model and support the selected optimal conditions for future scale-up processes.

#### 3.2.1 Reduction of Doping Co-solvent Stream: Bench Scale Validation

Addressing processing at a larger scale, a reduction in the co-solvent stream (EtOH) is proposed in order to minimize the use of organic solvents. Although EtOH is classified as a GRAS, non-toxic agent, the handling of kilograms of a flammable solvent is not compatible with green chemistry practices, with greater emphasis at higher levels of operation. In addition, its presence in the final product can hinder further utilization of the targeted extract and its applications in diverse fields. Thus, an adjustment in the process efficiency was pursued to obtain greener and scalable processes.

On this basis, greener practices were adopted, reducing the cosolvent stream to 4% and 2% of the total flow. For the latter, a bench scale level (35 g) was considered in order to move toward pilot-scale operations. Thus, an increased total flow (40 mL/min) was required, while the rest of the parameters were maintained. A summary that includes defatting, oil, and extraction performance, experimental conditions, and specifications is reported in Table 2.

**Table 2:** Extraction, Oil, and Defatting Yield (%) Achieved Through Supercritical CO_2_ Extraction of persimmon peels Combined With Fruit Apex Residues (PPsAF) at Various Levels of Operation (LoOP)^a^

supercritical CO_2_ device	lab-scale LoOP	oil yield (%)	defatting yield (%)	extraction yield (%)
10%-ethanol-doped	minor (3.5 g)	10.3 ± 0.7	82.9 ± 5.6	23.9 ± 7.9
4%-ethanol-doped	minor (3.5 g)	1.8 ± 0.7	14.8 ± 6.0	8.2 ± 1.1
2%-ethanol-doped	bench (35 g)	0.25 ± 0.07	1.8 ± 0.4	2.9 ± 0.3

a Experimental conditions: 35.2 MPa pressure; 50.1 °C temperature; 120 min time.

As can be observed in Table 3, an EtOH stream reduction in the Sc-CO_2_ phase also entails a decrease in extraction, oil, and defatting yields. Nonetheless, various aspects should be discussed at this point to adjust the maximum efficiency to a larger scale. For instance, although the highest doping level (10%-EtOH) leads to an acceptable performance, especially in terms of defatting (82.9%), this solid:solvent ratio cannot be assumed in industrial operations. Moreover, EtOH is normally injected as a fresh stream for each experiment at the industrial level, so its handling and cost are not acceptable. Therefore, other alternatives should be considered for implementing the methodology on a large scale.

**Table 3:** Total Carotenoids (TC), Antioxidant Capacity (TAC), and Phenolic Content (TPC) of Persimmon Peels Combined With Fruit Apex Residues (PSsAF) Extracts Recovered at Various Levels of Operation (LoOP) via Supercritical CO_2_

supercritical CO_2_ device	lab-scale LoOP	TC (μg/g extract)	TAC (μmol TE/g extract)	TPC (mg GAE/g extract)
10%-ethanol doped	minor (3.5 g)	27.25 ± 1.00	3.21 ± 0.95	4.09 ± 0.16
4%-ethanol doped	minor (3.5 g)	38.93 ± 1.78	5.41 ± 0.27	9.48 ± 1.20
2%-ethanol doped	bench (35 g)	226.94. ± 18.25	7.20 ± 0.30	33.52 ± 2.25

As mentioned, a reduction in doping was applied (2–4% EtOH), with a marked decrease in extraction (2.9–8.2%), oil yield (0.25–1.5%), and defatting (1.8–14.8%). It can be associated with solubility issues, which can be possibly attributed to the presence of carotenoids and/or polyphenols in persimmon materials. Thus, Sc-CO_2_ is more prone to remove non-polar components, such as fatty acids, so that a higher polar stream would favor other components present in PSsFA, such as carotenoids or polyphenols, as referred. At this point, the assessment of the nutritional and bioactive fractions of the extracts is crucial to determine the optimal conditions at a larger LoOP.

#### 3.2.2 Biological Assessment

Persimmons (*D. kaki*) are a considerable source of carotenoids and polyphenols, which have been shown to have protective effects against UV radiation in bacteria, fungi, algae, and plants. In addition to their protective role, these phytochemical compounds have antioxidant, anti-inflammatory, or antimicrobial properties.^11^ In this work, a preliminary biological assessment that includes total carotenoids (TC), antioxidant capacity (TAC), and phenolic content (TPC) of the PSsFA extracts recovered at lab scale has been carried out (Table 3). These outcomes will serve as a basis to select the adequate conditions for a further pilot-scale extraction.

Table 3 confirms that different EtOH doping ranges in the Sc-CO_2_ phase have a noted influence on the total content of the targeted compounds. By comparing 4–10% experiments, no significant differences were appreciated for TC, TAC, and TPC (27–39 μg/g extract, 3.21–5.41 μmol TE/g extract, and 4.09–9.48 mg GAE/g extract, respectively), albeit 4% seems to be slightly more effective to the targeted compounds regardless the lower oil and defatting yield (Table 2). That highlights the added value of this targeted fraction compared to 10%-EtOH system. In addition, a higher EtOH load could favor the extraction of polyphenols, competing with carotenoids and resulting in a lower concentration. A percentage of 10% could modify the physical characteristics of Sc-CO_2_, such as its density and viscosity, thus reducing the total carotenoid content in the extract. Thus, this polarity alters the solubility of bioactive compounds in the plant matrix.^28^ On the other hand, scCO_2_ density has an appreciable impact on both extraction yield and the amount of carotenoids extracted.^29^ Therefore, the use of a 4% doping has a minimal impact on the properties of Sc-CO_2_, enabling a major recovery of carotenoids.

Following this trend, outcomes displayed by the 2%-EtOH system were particularly noteworthy, with a TC content of 226.94 μg/g extract, 7.2 μmol TE/g extract, and a TPC of 33.52 mg GAE/g extract (Table 3 **, entry 3**). These enhanced values above 10- and 4%-EtOH-doped systems, which showed close values of 27–39 μg/g extract for TC, 3.21–5.41 μmol TE/g extract for TAC, and 4.09–9.48 mg GAE/g extract for TPC, demonstrate the added value of this fraction. Thus, despite the low defatting accomplished because of the low extract recovered, the bioactive power of this fraction is prominent. Namely, TC content displayed 10-fold compared to the initial 10%-EtOH system, as well as TPC achieving 33.52 mg GAE/g extract (8 times more *vs*. the initial). With these lectures, it is observed that a higher extraction and defatting yield is not related to a higher bioactivity in the recovered extract. This fact points out the value of the recovered fraction through a 2% doping that shows a superior quality.

Overall, the process is validated through a bench-scale strategy (35 g, 40 mL/min, 2% doping) that affords a distinguished bioactivity for TC, TAC, and TPC while promoting greener practices by reducing the organic solvent stream. The methodology will be thus implemented at the pilot level to corroborate the efficiency of the Sc-CO_2_ technology to recover the bioactive fraction and nutrients of PSsFA.

At this point, it should be mentioned that experiments were conducted at bench scale following the same LoOP, although no doping in the supercritical phase was adopted (i.e., 0%). In this regard, the release of the extract was troublesome, and no extract was recovered in the collection bottle due to the experimental conditions, Sc-CO_2_ system, or the nature of the PSsFA, with a low proportion of fat that is more suitable for removal with a pure Sc-CO_2_ stream. Thus, no reliable conclusions were obtained, and no data were reported.

### 3.3 Pilot Scale Extraction

#### 3.3.1 Process Efficiency and Biological Assessment

A pilot scale process (2 kg) was pursued in order to go toward industrial level. A residual doping (2%) was adopted as the main device based on the above reported optimization that concluded 35.2 MPa pressure and 50.1 °C temperature as the optimal conditions. Nevertheless, two proof-of-concepts that include (i) no doping in the Sc-CO_2_ stream and (ii) 4%- EtOH modification were conducted for the sake of comparison. The reason was to prove the methodology developed at lab-scale at different LoOP based on a balance between process efficiency, a noted bioactivity of the recovered oils, and green chemistry practices, as mentioned. In particular, an enhanced driving force was required for the 0%-doping extraction to facilitate the release of the extract, so that experiments were conducted at the maximum pressure allowed (40 MPa). Extraction, oil, and defatting yield (%), mass balance, and TC, TAC, and TPC are displayed in Table 4. Both extraction products are shown in Figure 2.

**Table 4:** Mass Balance for Pilot Scale Extraction of persimmon peels Combined With fruit apex Residues (PSsAF) Through Supercritical CO_2_ ^a^

	0%-EtOH-doped	2%-EtOH-doped	4%-EtOH-doped
**m**_**o**_ **(g)**	1800.0	1999.4	1000.1
**m**_**f**_ **(g)**	1746.6	1984.4	969.2
**extraction yield (%)**	3.0	0.8	3.1
**m**_**crude**_ **(g)** ^b^	–	3732.2	7466.1
**m**_**oil**_ **(g)** ^c^	25.1	39.1	35.1
**oil yield (%)**	1.4	2.0	3.5
**defatting yield (%)**	11.2	15.8	28.3
**TC (μg/extract)**	traces	1.87 ± 0.01	13.78 ± 0.80
**TAC (μM TE/g extract)**	0.72 ± 0.16	1.03 ± 0.09	0.60 ± 0.13
**TPC (mg GAE/g extract)**	4.79 ± 0.45	4.42 ± 0.78	2.57 ± 0.55

a Experimental conditions: 35.2 MPa pressure and 50.1 °C temperature (2–4%-EtOH) or 40 MPa pressure and 50.1 °C temperature (0%-EtOH extraction). Abbreviations: EY: extraction yield; OY: oil yield; DY: defatting yield.

b It refers to the total mass of crude recovered after pilot-scale extraction, including fresh ethanol.

c It refers to the mass of crude oil recovered after ethanol removal. TC: total carotenoid. TAC: total antioxidant capacity. TPC: total phenolic compounds.

**Figure 2:**
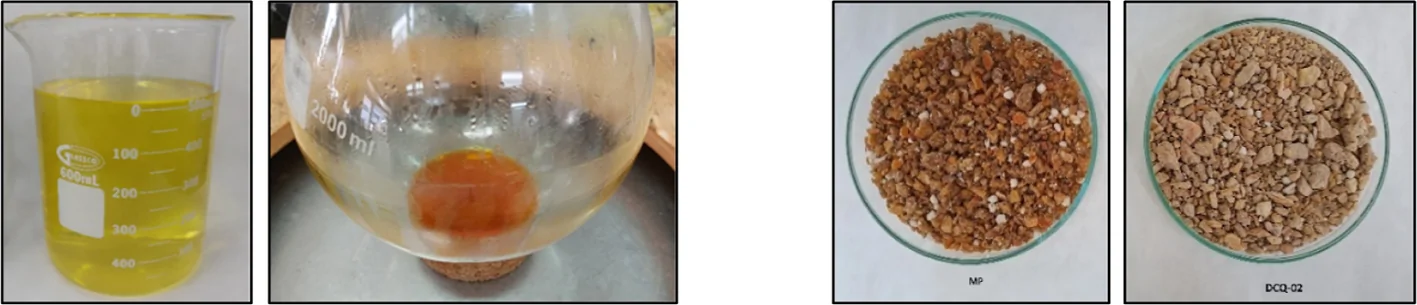
Raw and dried PSsAF crudes recovered through scCO_2_ at pilot plant (left); raw and post-treatment PSsFA solid recovered in this extraction (right) (free domain).

As referred previously, a balance between process efficiency, high bioactivity, and green chemistry should be considered. As deemed by the authors, this equilibrium is achieved by conducting the extraction at 2%-EtOH doping, in line with the optimized system at lab- and bench scale. This device entails a 2% and 15.8% oil and defatting yield, respectively, with close 1% extraction yield. In addition, the bioactivity is acceptable, showing 4.42 mg GAE/g extract for TPC, a TAC of 1.03 μm TE/g extract, and a TC content of 1.87 μg/g extract.

Although a 4% doping leads to the highest oil and defatting yield (3.5 and 28.3%, respectively) and TPC (13.78 mg GAE/g extract), the required amount of EtOH to execute the process is more than significant, with 7.5 kg recovered in the final crude. This volume is excessive to operate at an industrial level, even more knowing that EtOH is a highly flammable solvent. In addition, only 1 kg of PSsFA can be processed under this system in order to maintain an acceptable solid:liquid ratio at a large level. That represents a real drawback in the search for a higher LoOP to valorize biomass. Therefore, experiments at 4% doping were ruled out from this point onward.

In the absence of doping (i.e., 0%-EtOH), the lowest defatting was accomplished (11.2%), which relates to 1.4% oil yield. Regardless of having similar values as 2% extraction (15.8% defatting and 2% oil), the bioactivity in the crude was reduced compared to the 2–4% experiments, especially for TC, where only traces were found. Moreover, these experiments had to be conducted at the highest pressure allowed by the system (40 MPa) since in the absence of co-solvent the extraction of the oil can be hindered. Regardless, no significant improvement has been found.

#### 3.3.2 Green Chemistry Metrics

A pilot-scale process was executed in order to test the real scope of the methodology developed at lab scale at different LoOPs. At this point, the environmental sustainability of the process can be evaluated through different metrics related to the green chemistry indicators. Complete environmental factor (CEF), process mass intensity (PMI), process mass productivity (PMP), reaction mass efficiency (RME), or eco-scale were considered (Figure 3). Green metrics were calculated attending to the oil and defatting yield (%) for pilot-scale operations at 2%-EtOH-doped, given the above conclusions about mass balance, biological activity, and green chemistry. The methodology for calculations was based on previous studies that also tackled a green chemistry assessment with these metrics,^25^ albeit the protocol was adapted to this work (Supporting Information, section S3). A bench-scale process was also evaluated for the sake of comparison (Figure 3).

**Figure 3:**
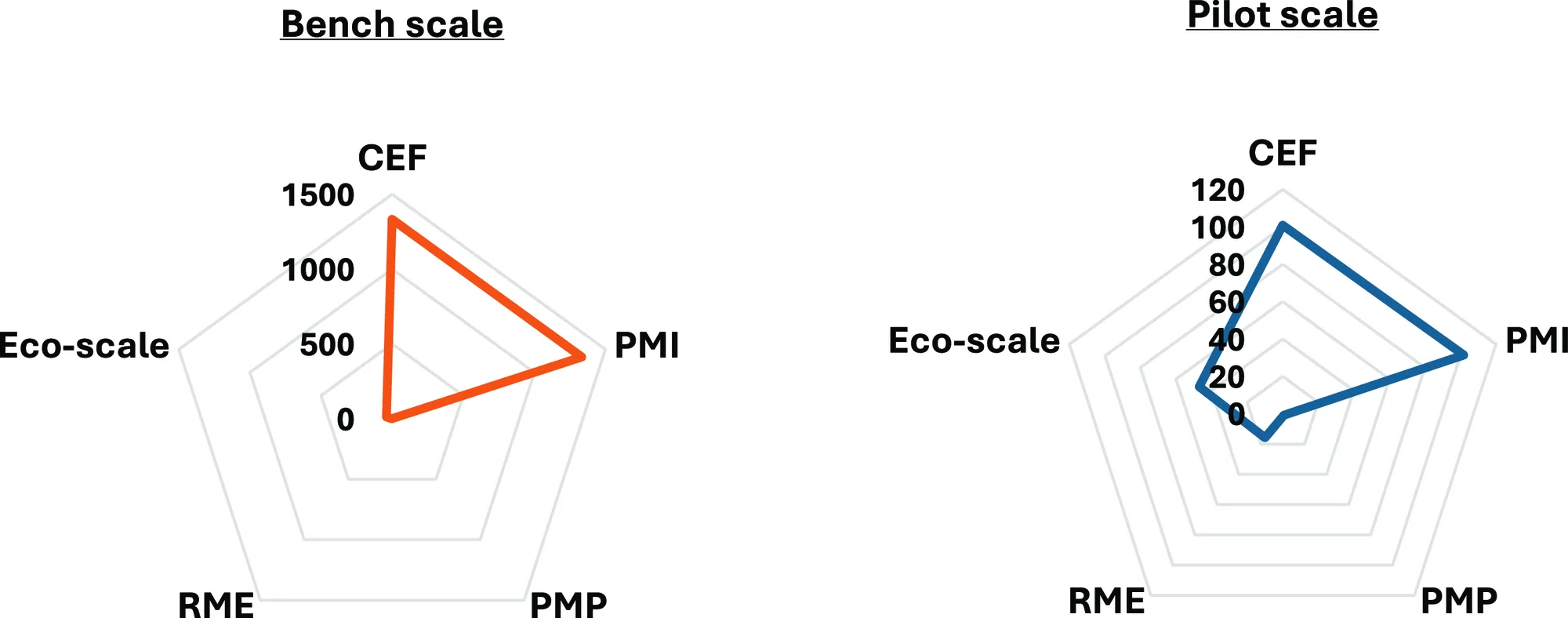
Green chemistry metrics for the scCO_2_ process at bench- and pilot-scale.

As observed in Figure 3, the results obtained are far from the most desirable from a green chemistry point of view. This fact is mainly attributed to the moderate amount of oil recovered (only a few grams of 2 kg of raw material). It entails the generation of a high amount of waste, which is mainly related to CEF. In addition, the high mass of EtOH also affects the PMI, which includes the total mass of agents involved in the process. In this regard, these two metrics (CEF and PMI) lead the bench-scale process, which showed very poor values for PMP (0.075%) and RME (1.4%). Only the eco-scale, which constitutes a scale from 0 to 100 (the most benign process), showed acceptable values, albeit still below 50 (39.7). The reason is the different cumulative penalty points (CPPs) attributed to the unconventional activation techniques, high pressures, low yields, or flammability of EtOH, according to the CPP scale proposed by Van Aken et al.^30^

Besides, pilot-scale operations considerably attenuated the environmental footprint compared to bench-scale processes, as could be expected. Albeit CEF and PMI still set the trend in the metrics, a 10-fold reduction is achieved compared to the BS process (CEF: 1332.8 at BS and 100.8 for PS; for PMI: 1333.8 at BS and 101.8 at PS). Nonetheless, PMP and RME still showed very low values (1 and 15.9%, respectively). Finally, eco-scale situated in a mark of 45.9, slightly higher than BS process attending to the reduction in CPP as a consequence of the higher defatting yield.^30^

To sum up, these metrics show the need to still adequately the process at an industrial level and to enhance process efficiency. Nevertheless, the methodology employs substances compatible with green chemistry (i.e., non-toxic, non-harmful, or non-hazardous) since only CO_2_ and EtOH intervene in the extraction. In addition, the process adopts practices in line with carbon capture and utilization (CCU), enabling the utilization of CO_2_ as an extraction medium above traditional organic solvents. Therefore, this study demonstrates the feasibility of Sc-CO_2_ technology and provides novel advances in relation to the persimmon cull processing at a large scale.

### 3.4 Enhanced Biological Performance and Nutritional Indicators: Mineral Profile and Antimicrobial Activity (Pilot-Scale Extract)

Despite the above green chemistry evaluation and the moderate yields accomplished, a valuable product that showed biological and nutritional activity according to its TC, TAC, and TPC values was obtained. To deeply study this crude and point out its exceptional properties, the mineral profile was assessed in parallel to its antimicrobial activity vs different bacteria, yeast, and filamentous fungi. For these analyses, and based on the reported conclusions, the extract recovered at 2%-EtOH-doped extraction at pilot level was considered.

#### 3.4.1 Mineral Profile

The mineral profile of the PSsFA extract recovered through Sc-CO_2_ at pilot scale is shown in Table 5. Furthermore, to deeply evaluate the nutritional quality of the extract, a comparison to the daily recommendations established by the European Food Safety Authority (EFSA) in 2017 ^31^ was carried out. Thus, the percentage of Dietary Reference Intakes (RDIs) covered by these minerals is assessed (Table 5).

**Table 5:** Daily Recommendations Established by the EFSA^31^ for the Minerals Analyzed in the Persimmon Extract Recovered via Supercritical CO_2_

mineral	persimmon extract (mg/g)	RDI (mg/100 g extract)	RDI (mg/day)^a^	% RDI
**Mg**	1.81 ± 0.0004	181	300–350	51.7%
**P**	0.723 ± 0.008	72.3	550	13.2%
**K**	7.77 ± 0.15	777	3500	22.2%
**Ca**	0.562 ± 0.007	56.2	950	5.9%
**Fe**	0.858 ± 0.013	85.8	11–16	536.2–780%
**Cu**	0.00192 ± 0.00003	0.19	1.3–1.6	11.9–14.6%
**Zn**	0.00486 ± 0.00012	0.486	9.3–11.7	4.1–5.2%

a Data reported for men and women between 20–60 years of age, based on the RDI provided by EFSA. An inferior value in the reported intervals corresponds to women data in any case.

Overall, persimmons pose a more than interesting nutritional value based on their balanced mineral content reported in Table 5. That contributes to an adequate intake of essential mineral elements for the prevention of nutrition-associated chronic and degenerative diseases, such as cancer, cardiovascular disease, or obesity.^32^

First, it should be highlighted that the iron content is 0.86 mg/g extract. This value represents the intake of up to 85.8 mg/100 g extract, which corresponds to a superior RDI (536.2–780%) compared to recommended values of EFSA (11–16 mg/day). Therefore, the persimmon crude being extracted through a Sc-CO_2_ system on a large scale emerges as a potential iron supplement. That is important since iron is involved in several metabolic processes in humans, including DNA synthesis, and electron and oxygen transport.^33^

Magnesium, an essential mineral that is mostly found in bones and teeth (70%), but also in soft tissues such as muscles, heart, liver, pancreas, and external fluids, is also of relevance. Its intake is important in the growth of children, pregnant, lactating, or menstruating women, and in the elderly. Further, it is also involved in cellular metabolism and up to 300 enzyme systems.^34^ As observed in Table 5, the intake of 1 g of persimmon extract is related to 1.81 mg Mg, which means 181 mg in each 100 g. The RDI achieves 52% of the Mg levels recommended by the EFSA for 20–60 years old men and women (300–350 mg/day).

Other major minerals include phosphorus, potassium, and calcium. As Table 5 displays, the intake of 100 g of persimmon extract would correspond to an RDI ranging between 5.9% (Ca) to 22.2% (K) of the recommended values (Table 5). Regardless of not being the most desirable values, a moderate amount was detected in this crude, especially for potassium. Therefore, the relevance of these minerals should be pointed out to determine whether an appropriate use in novel food formulations is expected. For instance, phosphorus forms part of bones, teeth, DNA, and RNA, and it is involved in the synthesis of various enzymes as well as mono-, di-, and adenosine triphosphates (AMP, ADP, ATP). In addition, it plays a key role in the regulation of gene transcription, enzyme activation, maintenance of normal pH in the extracellular fluid, and intracellular energy storage.^34^ By contrast, potassium regulates the activity of the heart, muscles, nervous system, and most cellular functions. It also assists in the transmission of nerve impulses and participates in the contraction of the heart muscle.^35^ Finally, calcium provides strength and rigidity to the skeletal structure. It also participates in the regulation of heart rate, nerve transmission, muscle contraction, and blood clotting, as well as in the control of the acid–base balance of the blood.^34^

Besides, minor minerals such as copper or zinc are not so relevant in persimmons, with RDI between 4.1–14.6% for the intake of 100 g of extract and values far from the recommended values. In any case, a biological involvement of these minerals should be highlighted: copper is used to produce energy and form connective tissues or blood vessels. In addition, it helps to maintain the proper functioning of the nervous and immune system while participating in gene activation.^36^

Further, zinc is necessary for the catalytic activity of hundreds of enzymes and contributes to the improvement of immune function, protein and DNA synthesis, wound healing, and cell signaling and division. It also supports healthy growth and development during pregnancy, childhood, and adolescence and contributes to the sense of taste.^34^

In summary, the persimmon lipophilic extract recovered through a pilot-scale Sc-CO_2_ extraction is supposed to be a prominent source of iron (0.858 mg/g extract). While relevant values were found for magnesium (52% RDI) and potassium (22% RDI), which points out the nutritional value of this extract. These achievements make the product a promising candidate for further investigations in the search for novel nutritional formulations that could fill the nutritional needs of different population groups, contributing to their health and well-being. Nevertheless, to confirm these outcomes, further research is required to ensure efficacy and safety as well as to determine its appropriate dosage.

#### 3.4.2 Antimicrobial Evaluation

To further support the nutritional importance of the persimmon extract based on the above results, the *in vitro* antimicrobial activity was also assessed against different microorganisms: the bacteria *B. subtilis* and *E. coli*, selected for their relevance in food spoilage^37^ and foodborne illness outbreaks;^38^ the yeasts *S. cerevisae* and *Z. bailii*, for their frequent occurrence as undesired food contaminants;^39^ and the filamentous fungi *P. expansum* and *A. flavus*, both of which are important mycotoxin producers^40,41^ (Table 6; Figure S2). Oregano oil was used as a positive control due to its well-documented antimicrobial efficacy against a wide range of microorganisms.^42^

**Table 6:** Antimicrobial Activity (μL/mL) of Persimmon Lipophilic Extract Recovered via Supercritical CO_2_

	microorganism	persimmon crude		oregano oil	
		MIC	MLC	MIC	MLC
bacteria	*B. subtilis* CECT 498	0.8	NA^a^	0.8	6.3
	*E. coli* JM109	3.1	12.5	0.8	6.3
yeasts	*S. cerevisae* BY4741	>2.0	ND^b^	2.0	ND
	*Z. bailii* J81	>2.0	ND	2.0	ND
filamentous fungi	*P. expansum* A72	7.8	7.8	7.8	7.8
	*A. flavus* RB9	31.3	NA	31.3	31.3

a NA: no killing activity at the conditions tested.

b ND: not determined.

Persimmon extract exhibited antimicrobial activity with MIC values of 0.8 μL/mL against *B. subtilis*, 3.1 μL/mL for *E. coli*, 7.8 μL/mL for *P. expansum*, and 31.3 μL/mL for *A. flavus* (Table 6; Figure S2), while no complete inhibitory effect was detected against yeasts under the conditions tested. In comparison, oregano oil showed stronger antimicrobial activity against bacteria and yeasts, with MIC values of 0.8 μL/mL against both *B. subtilis* and *E. coli*, and 2 μL/mL against *S. cerevisae* and *Z. bailii*. In case of filamentous fungi, both oils showed identical MIC values: 7.8 and 31.3 μL/mL against *P. expansum* and *A. flavus*, respectively.

When a MIC was detected in the case of persimmon crude, additional assays were performed to determine the MLC. This extract exhibited bactericide activity against the Gram-negative *E. coli* at 12.5 μL/mL, but not against the Gram-positive *B. subtilis*.

In contrast, oregano oil showed stronger bactericide activity, killing both bacterial strains at 6.3 μL/mL. In case of filamentous fungi, the persimmon-derived crude exhibited lethal activity against *P. expansum* at 7.8 μL/mL, but not against *A. flavus*, whereas oregano oil killed both *P. expansum* and *A. flavus* at 7.8 and 31. 3 μL/mL, respectively.

The lower MIC of persimmon extract against the Gram-positive strain *B. subtilis* compared to the Gram-negative *E. coli* is consistent with the general notion that Gram-positive bacteria are more susceptible to essential oils, mainly due to the absence of an outer membrane that acts as a permeability barrier in Gram-negative bacteria.^43^ Interestingly, this pattern was not observed with oregano oil, which maintained high efficacy against both bacterial groups, suggesting the presence of bioactive compounds with stronger mechanisms of action, such as carvacrol.^44^

In general, the MIC of the tested antimicrobial agents tends to increase with taxonomic complexity: bacteria exhibited the lowest MICs, whereas filamentous fungi exhibited the highest tolerance under comparable testing conditions. This is in agreement with previous studies highlighting that filamentous fungi often require higher antimicrobial concentrations due to their morphological characteristics, such as hyphal networks, which may hinder the direct interaction of the oil components with cell membranes.^45^

Overall, these results indicate that persimmon lipophilic extract possesses promising antimicrobial activity, particularly against *B. subtilis*, *E. coli*, and *P. expansum*. While its potency is lower compared to oregano oil, its activity against relevant food-contaminating microorganisms suggests potential applications as a natural preservative. Although the compounds responsible for this activity were not identified in this study, previous studies have shown that compounds such as those present in persimmon-derived extracts contain bioactive molecules with reported antimicrobial properties, including carotenoids with microbial anti-adhesive effects,^46^ and phenolic compounds,^47^ which may contribute individually or synergistically to the observed effects. These compounds are present in the persimmon extract analyzed in a concentration of 1.87 μg/g extract (total carotenoids) and 4.42 mg GAE/g extract (phenolic compounds) (Table 4). However, future studies focusing on the chemical characterization of persimmon lipophilic extract constituents could shed light on the mechanisms underlying its antimicrobial activity.

## 4 Conclusions

A greener methodology that employs Sc-CO_2_ as a process intensification strategy has been applied in this work to tackle a chemical valorization of persimmon side-streams. Namely, a pilot-scale extraction (2%-ethanol-doped, 2 kg of sample) enabled the production of a bioactive, nutritionally promising candidate for further investigations in the search for novel nutraceutical products. In particular, this lipophilic extract showed an important iron and magnesium content (85.8 mg/100 g extract and 530–780% RDI for Fe; 181 mg/100 g extract and 52.7% RDI for Mg), as well as an acceptable antimicrobial activity against relevant foodborne microorganisms, such as *B. subtilis*, *E. coli*, or the mycotoxin-producing fungus *P. expansum*. A sustainability assessment of the methodology revealed that the environmental footprint was considerably reduced at the pilot level compared to bench scale experiments, with a 45.9 score in the eco-scale. Overall, the work paves the way for novel antimicrobial and food solutions from discarded materials via process intensification, which aligns with biorefinery principles.

## Supplementary Material



## Notes

SECTION S1: FIGURES. Figure S1. Optimal conditions estimated by the design response–surface (a), and effect of pressure, temperature and time variables on oil yield (b); Figure S2. Antimicrobial activity of persimmon lipophilic extract against bacteria (A), yeasts (B) and filamentous fungi (C). Dose–response curves show the mean ± standard deviation (SD) of the percentage of growth from triplicate samples. Measurements were taken after 48 h of incubation at 37 °C for bacteria, 30 °C for yeasts, and 72 h at 25 °C for filamentous fungi; SECTION S2: TABLES. Table S1. Time (min), temperature (°C) and pressure (MPa) conditions for the 15 experiments included in the optimization through the response-surface design; SECTION S3**:** METHODOLOGY FOR GREEN CHEMISTRY METRICS CALCULATION (WORD FILE). (PDF)
