# Supplementary material for: Ethanol-Modified, Supercritical CO2 Extraction of Persimmon (Diospyros kaki L.) Side Streams for Innovative Bioactive and Antimicrobial Formulations: A Pilot Level Proof-of-Concept and Green Chemistry Evaluation
Source: ACS Omega. 2026 Sep 16;11(38):57269–80. doi: 10.1021/acsomega.6c05435 (PMC13629406; doi:10.1021/acsomega.6c05435)
Supplement: Supplementary file 1 [file ao6c05435_si_001.pdf]

Ethanol-modified, supercritical CO_2 extraction of persimmon (*Diospyros kaki* L.) side streams for innovative bioactive and antimicrobial formulations: A pilot level proof-of-concept and Green Chemistry evaluation

Albert Sebastià[1], Manuel Salgado-Ramos[1, *], Noelia Pallarés[1], Samantha López-Contreras[2], Sandra Garrigues[2], Juan A. Tamayo-Ramos[2], Raquel Lucas-Gonzalez[3], Juana Fernández-López[3], Francisco J. Barba[1,a]

[1] Research group in Innovative Technologies for Sustainable Food (ALISOST), Department of Preventive Medicine and Public Health, Food Science, Toxicology and Forensic Medicine, Faculty of Pharmacy and Food Sciences, Universitat de València, Avda. Vicent Andrés Estellés, 22, 46100 Burjassot, Valencia, Spain

[2] Food Biotechnology Department, Instituto de Agroquímica y Tecnología de Alimentos (IATA), Consejo Superior de Investigaciones Científicas (CSIC), Valencia, Paterna, Spain

[3] Research group in Innovations in Food Products (IPOA), Institute for Agri-Food and Agri-Environmental Research and Innovation, Miguel Hernández University (CIAGRO-UMH), Ctra. Beniel km 3.2, 03312 Orihuela, Alicante, Spain

[*] Correspondence should be addressed to:

Dr. Manuel Salgado Ramos

[a] Additional address: CIBER de Enfermedades Infecciosas (CIBERINFEC), Instituto de Salud Carlos III, C/ Monforte de Lemos 3-5. Pabellón 11. Planta 0, 28029 Madrid, Spain

email: manuel.salgado-ramos@uv.es

Supplementary material

SECTION S1. FIGURES

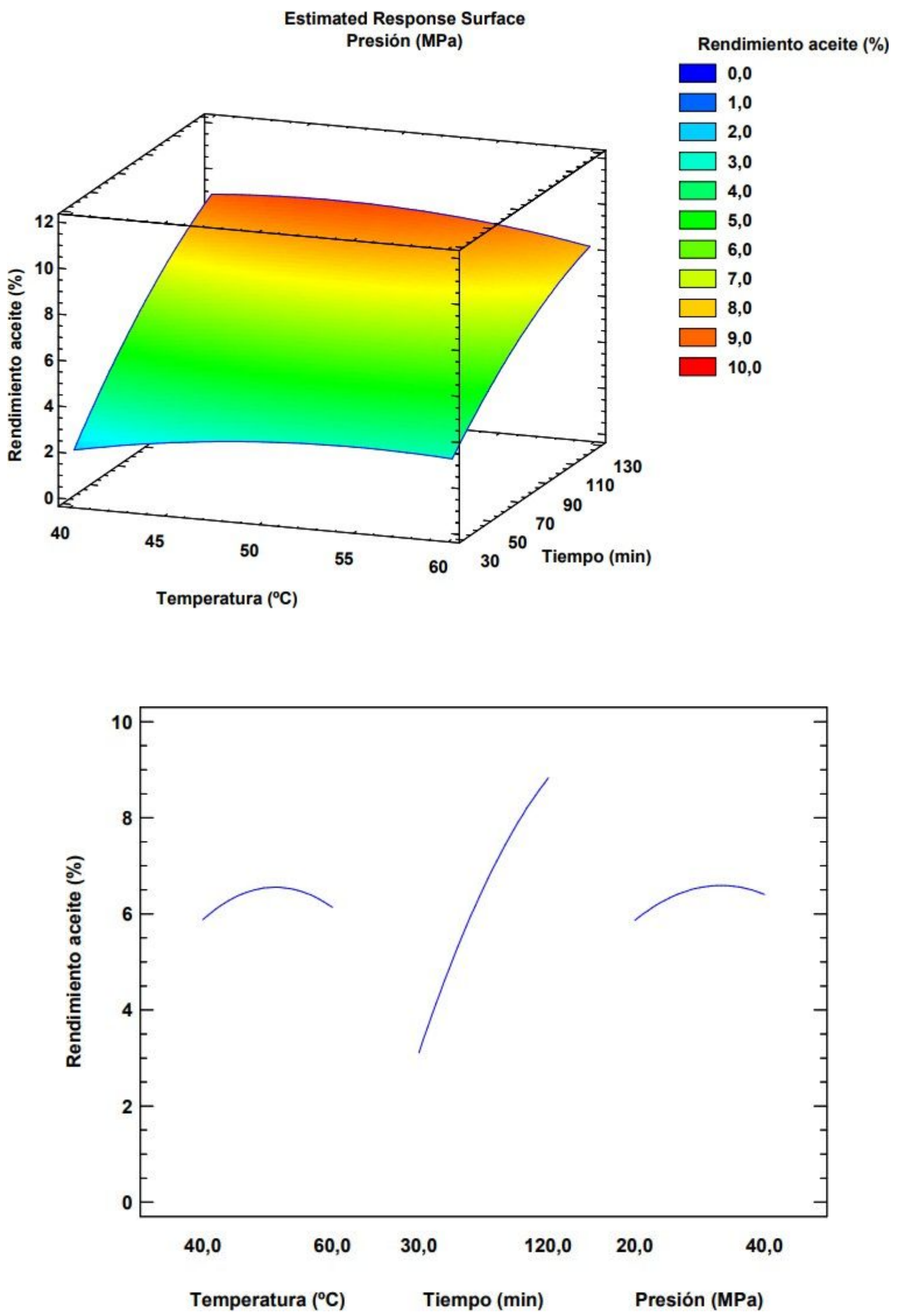

Figure S1. Optimal conditions estimated by the design response – surface (a), and effect of pressure, temperature and time variables on oil yield (b).

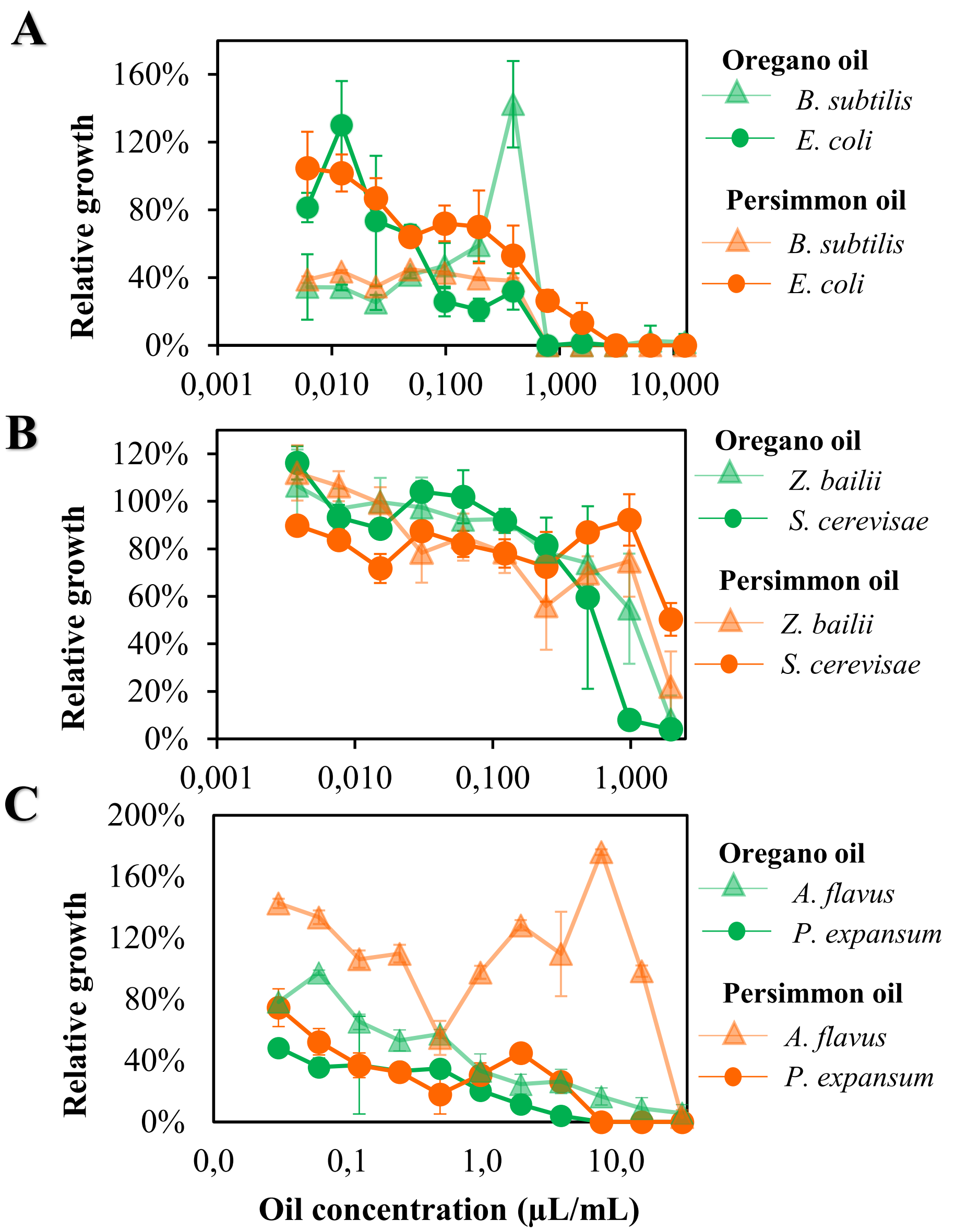

Figure S2. Antimicrobial activity of persimmon lipophilic extract against bacteria (A), yeasts (B) and filamentous fungi (C). Dose-response curves show the mean ± standard deviation (SD) of the percentage of growth from triplicate samples. Measurements were taken after 48 h of incubation at 37 °C for bacteria, 30 °C for yeasts, and 72 h at 25 °C for filamentous fungi.

SECTION S2. TABLES

Table S1. Time (min), temperature (ºC) and pressure (MPa) conditions for the 15 experiments included in the optimization through the response-surface design.

	T (°C)	t (min)	P (MPa)	Oil yield (%)	Defatting (%)
1	40	75	20	2.3	21.9
2	60	120	30	8.0	77.7
3	50	75	30	6.5	63.1
4	50	120	20	8.0	77.7
5	60	75	20	4.9	47.2
6	50	30	20	3.1	30.2
7	50	120	40	8.3	80.5
8	40	75	40	5.1	50
9	50	75	30	6.0	58.3
10	50	30	40	2.8	27.6
11	60	30	30	2.5	25.1
12	40	30	30	2.0	19.6
13	50	75	30	7.1	69.4
14	60	75	40	7.1	69.4
15	40	120	30	9.0	73.7

SECTION S3. METHODOLOGY FOR GREEN CHEMISTRY METRICS CALCULATION

The environmental footprint associated with the extraction processes at bench and pilot scale (**BS** and **PS**, respectively) was assessed by Green Chemistry Performance Metrics (GCPMs). Complete Environmental factor (CEF), Process Mass Intensity (PMI), Process Mass Productivity (PMP), Reaction Mass Efficiency (RME), and Eco-Scale were the main metrics considered in this work.

Based on the optimal conditions found, experiments conducted at 2%-ethanol-doped were included this assessment.

Firstly, the mass balance for the extraction processes conducted is detailed in **Table S2**:

Table S2. Mass balance for $scCO_2$ extraction of PsSFA at bench- and pilot scale (BS and PS) at 2%-ethanol-doped.

	PsSFA	Initial lipophiles content involved (g)[a]	EtOH (g)[b]	Defatted PsSFA (g)	Recovered oil (g)
**BS**	35	4.3	75.7	31	0.06
**PS**	1999.4	247.9	3732.2	1984.4	39.1

a. Calculated by PsSFA (g) x 0.124 (% of lipophiles in the raw material).
b. ρ_{EtOH}: 0.789 g/ml.

Considerations for GCPMs calculation:

i) Uniquely, the oil recovered after SFE process is the desirable product. For this methodology, conversion process is defined as:

$$\textbf{PsSFA (s) + } \mathbf{CO_2} \textbf{ (sc) + EtOH (l)} \rightarrow \textbf{Defatted (D)-PsSFA (s) + oil (l)}$$

where only the lipophiles fraction involved in the raw PsSFA is considered. Regardless of D-PsSFA is a valuable product that can be exploited in a biorefinery context, it is not deemed a profitable material in this green chemistry assessment. However, it was neither considered as a real waste.

ii) Attending to the potential and feasibility of $scCO_2$ at industrial scale, it is well-known the wide range of technological solutions that are implemented in the industry, not only in relation to CO_2 transport and storage but also recycling. Considering this issue, CO_2 was ruled out for calculations of GCPMs.

iii) Contrarily to CO_2, EtOH was indeed included for green metrics calculations due to its high flammability, special management at high level, and the important loss that occurred by volatilisation or due to the amount that remain in the defatted material. In addition, fresh streams are introduced in the system for each experiment.

EtOH consumption at BS was calculated for a 120 min extraction. Contrarily, 5h were required at PS to achieve maximum efficiency and processing.

The below metrics have been carefully evaluated, based on the arguments above explained.

1. ENVIRONMENTAL FACTOR (E-factor): this metric evaluates the amount of waste generated throughout a chemical process:

$$\mathrm{E-factor} = \frac{\text{mass of waste}}{\text{mass of product}}$$

In this work, to evaluate the real impact of using organic solvents, **complete E-factor (CEF)** has been considered. So, the previous expression can be converted to:

$$\mathrm{CEF} = \frac{\text{mass of raw materials} - \text{mass of product}}{\text{mass of product}}$$

For these processes conducted at 2% doping, the mass of raw materials include both initial lipophiles content and ethanol. Thus, CEF is calculated by:

$$\mathbf{CEF\ (BS)} = \frac{4.3 + 75.7 - 0.06}{0.06} = \mathbf{1332.3}$$

$$\mathbf{CEF\ (PS)} = \frac{247.9 + 3732.2 - 39.1}{39.1} = \mathbf{100.8}$$

2. PROCESS MASS INTENSITY (PMI) evaluates the total mass of reagents and solvents utilised in the process with respect to target product:

$$\mathrm{PMI} = \frac{\text{total mass used in a process}}{\text{mass of product}}$$

According to the mass balance reported in **Table S2**, PMI is calculated as:

$$\mathbf{PMI\ (BS)} = \frac{4.3 + 75.7}{0.06} = \mathbf{1333.3}$$

$$\mathbf{PMI\ (PS)} = \frac{247.9 + 3732.2}{39.1} = \mathbf{101.8}$$

Otherwise, PMI can be obtained by: PMI = 1 + CEF.[3]

3. PROCESS MASS PRODUCTIVITY (PMP) measures the real productivity of the process and is calculated by:

$$\text{PMP (\%)} = \frac{\text{mass of product}}{\text{total mass used in a process}} \text{ x } 100 = \frac{1}{\text{PMI}} \text{ x } 100$$

For the experiments selected, PMP is obtained by:

$$\textbf{PMP}\ (\%)\ (\textbf{BS}) = \frac{1}{1333.3} \text{ x } 100 = \textbf{0.075}\ \%$$

$$\textbf{PMP}\ (\%)\ (\textbf{PS}) = \frac{1}{101.8} \text{ x } 100 = \textbf{0.98}\ \%$$

4. REACTION MASS EFFICIENCY (RME): this metric refers to the percentage of the reactants that are included in the product, and it is obtained by:

$$\text{RME (\%)} = \frac{\text{mass of product}}{\text{mass of reactants}} \text{ x } 100$$

As this metric principally evaluates a whole reaction process between reagents and products (i.e., the initial mass of lipophiles and the total mass of oil recovered), ethanol was ruled out from RME. Therefore, this metric follows the calculation below:

$$\textbf{RME}\ (\%)\ (\textbf{BS}) = \frac{0.06}{4.3} \text{ x } 100 = \textbf{1.4}\ \%$$

$$\textbf{RME}\ (\%)\ (\textbf{PS}) = \frac{39.1}{247.9} \text{ x } 100 = \textbf{15.8}\ \%$$

5. ECO-SCALE: this metric was planned by Van Aken *et al.*[30] and it uses a scale from 0 to 100 to indicate an eco-score, being 100 the most ecological process. This score comes from various cumulative penalty points (CPP) that consider yield, price of reaction

components, safety, technical requirements, heating and time, and/or purification.[30] CPP involved in all the processes developed are detailed in **Table S3**

Table S3 Cumulative penalty points (CPP) for the processes developed at bench and pilot scale (BS and PS, respectively).

Parameter	Process	CPP
**Yield**		
CPP = $\frac{100 - \%yield*}{2}$<br><br>* Defatting yield was considered<br>BS: 1.4 %<br>PS: 15.8 %	**BS**	49.3
	**PS**	42.1
**Price of reaction components**		
CO_2, EtOH: < 10$ (0 CPP)	**BS**	0
	**PS**	0
**Safety**		
- EtOH: highly flammable (5 CCP)<br><br>* Albeit CO_2 is dangerous for the environment and imply 5 CCP according to the scale, it was ruled out in GCPMs due to the possibility of recirculation.	**BS**	5
	**PS**	5
**Technical setup**		
- Instrument for controlled addition of chemicals: syringe pump (1 CPP) *<br>- Instrument for controlled addition of chemicals: gas pressure regulator (1 CCP)<br>- Unconventional activation technique ($scCO_2$) (2 CCP)<br>- Pressure equipment > 1 atm (3 CCP)<br><br>* 2% experiments require a syringe pump to inject EtOH.	**BS**	1 + 1 + 2 + 3
	**PS**	1 + 1+ 2 + 3
**Temperature / time**		
Heating > 1h (3 CPP) *<br><br>* Each extraction was conducted at 50°C, so CCP were not applied in this section based on the low temperatures applied.	**BS**	0
	**PS**	0
**Workup and purification**		
Removal of solvent with bp < 150°C (EtOH) (0 CPP)	**BS**	0

	**PS**	0

Attending to the CPP reported, eco-score can be calculated by:

$$\mathbf{Eco-scale\ (BS)} = 100 - 61.3 = \mathbf{38.7}$$

$$\mathbf{Eco-scale\ (PS)} = 100 - 54.1 = \mathbf{45.9}$$
